# Timing and distance of natal dispersal in Asian black bears

**DOI:** 10.1093/jmammal/gyac118

**Published:** 2023-02-14

**Authors:** Kaede Takayama, Naoki Ohnishi, Andreas Zedrosser, Tomoko Anezaki, Kahoko Tochigi, Akino Inagaki, Tomoko Naganuma, Koji Yamazaki, Shinsuke Koike

**Affiliations:** Faculty of Agriculture, Tokyo University of Agriculture and Technology, 3-5-8 Saiwai-Cho, Fuchu, Tokyo 183-8509, Japan; Tohoku Research Center, Forestry and Forest Products Research Institute, 92-25 Nabeyashiki, Morioka, Iwate 020-0123, Japan; Department of Natural Sciences and Environmental Health, University of South-Eastern Norway, N-3800 Bø in Telemark, Norway; Institute for Wildlife Biology and Game Management, University for Natural Resources and Life Sciences, Vienna, Gregor Mendel Str. 33, A-1180 Vienna, Austria; Institute of Global Innovation, Tokyo University of Agriculture and Technology, 3-5-8 Saiwai-Cho, Fuchu, Tokyo 183-8509, Japan; Gunma Museum of Natural History, 1674-1 Kamikuroiwa, Tomioka, Gunma 370-2345, Japan; Faculty of Agriculture, Tokyo University of Agriculture and Technology, 3-5-8 Saiwai-Cho, Fuchu, Tokyo 183-8509, Japan; Faculty of Agriculture, Tokyo University of Agriculture and Technology, 3-5-8 Saiwai-Cho, Fuchu, Tokyo 183-8509, Japan; Institute of Global Innovation, Tokyo University of Agriculture and Technology, 3-5-8 Saiwai-Cho, Fuchu, Tokyo 183-8509, Japan; Faculty of Regional Environmental Science, Tokyo University of Agriculture, 1-1-1 Sakuragaoka, Setagaya, Tokyo 156-8502, Japan; Institute of Global Innovation, Tokyo University of Agriculture and Technology, 3-5-8 Saiwai-Cho, Fuchu, Tokyo 183-8509, Japan; Institute of Agriculture, Tokyo University of Agriculture and Technology, 3-5-8 Saiwai-Cho, Fuchu, Tokyo 183-8509, Japan

**Keywords:** genetic structure, natal dispersal, parentage analysis, sex-biased dispersal, *Ursus thibetanus*

## Abstract

Dispersal has important implications for population ecology and genetics of a species through redistribution of individuals. In most mammals, males leave their natal area before they reach sexual maturity, whereas females are commonly philopatric. Here, we investigate the patterns of natal dispersal in the Asian black bear (*Ursus thibetanus*) based on data from 550 bears (378 males, 172 females) captured or removed in Gunma and Tochigi prefectures on central Honshu Island, Japan in 2003–2018. We used genetic data and parentage analysis to investigate sex-biased differences in the distance of natal dispersal. We further investigated the age of dispersal using spatial autocorrelation analysis, that is, the change in the correlation between genetic and geographic distances in each sex and age group. Our results revealed that male dispersal distances (mean ± *SE* = 17.4 ± 3.5 km) were significantly farther than female distances (4.8 ± 1.7 km), and the results were not affected by years of mast failures, a prominent forage source for this population. Based on an average adult female home range radius of 1.8 km, 96% of the males and 50% of the females dispersed. In the spatial autocorrelation analysis, the changes in the relationship between genetic and geographic distances were more pronounced in males compared to females. Males seem to mostly disperse at age 3 regardless of mast productivity, and they gradually disperse far from their home range, but young and inexperienced males may return to their natal home range in years with poor food conditions. The results suggest that factors driving the dispersal process seem to be population structure-based instead of forage availability-based. In females, a significant genetic relationship was observed among all individuals in the group with a minimum age of 6 years within a distance of 2 km, which resulted in the formation of matrilineal assemblages.

Dispersal has important implications for the population ecology and genetics of a species through redistribution of individuals (Clobert et al. 2001, 2009; Bowler and Benton 2005). Natal dispersal, defined as the movement of an individual from its birthplace to its breeding place (Howard 1960), is the most common mechanism of population redistribution, because subadults commonly move larger distances and at a larger proportion compared to adults in both birds and mammals (Cote et al. 2017). Dispersal can be of selective advantage when the fitness benefits of moving to a new patch exceed the costs of movement (Bowler and Benton 2005).

In mammals, there are commonly large differences between the sexes in relation to distances traveled during dispersal and dispersal rates (Lawson Handley and Perrin 2007). Several hypotheses have been proposed to explain sex-biased natal dispersal, such as inbreeding avoidance (Clutton-Brock 1989a), resource competition (Clark 1978; Greenwood 1980), local-mate competition (Dobson 1982), and kin cooperation (Greenwood 1980; Lawson Handley and Perrin 2007). Evidence suggests that each of these hypotheses may drive sex-biased dispersal, and that the influence of these drivers likely differs by species and system. In most mammals, males leave their natal range before they reach sexual maturity, whereas females commonly remain philopatric (Greenwood 1980; Pusey 1987). Sex-biased dispersal has been linked predominantly to polygynous mating systems, where local-mate competition for males exceeds local resource for females, and dispersal patterns depend on the costs and benefits of dispersal and philopatry for the inclusive fitness of individuals of each sex (Greenwood 1980; Pusey 1987; Lawson Handley and Perrin 2007). Mammals exhibit different social and mating systems, which has important implications for reproduction and resource acquisition, ranging from solitary to group-living, strict territoriality to overlapping home ranges, and strictly monogamous to polygamy (Clutton-Brock 1989b; Gittleman 1989; Klug 2011). Dispersal patterns are closely related to the social organizations of a species and may vary widely due to variations in selective pressure (Lawson Handley and Perrin 2007).

In general, dispersal is among the least understood behaviors of both individual animals and population dynamics (Sutherland et al. 2000), particularly in human-dominated landscapes (Reinhardt et al. 2019). Inferences of animal movements in the wild are commonly based on mark–recapture of known individuals (Bennetts et al. 2001; Nathan et al. 2008) or radiotelemetry (Cagnacci et al. 2010). An additional approach for inferring dispersal parameters is the use of genetic data, because such data can be obtained via noninvasive hair snare projects or from individuals found dead or removed from the population via hunting or due to control actions (Ross 2001; Rousset 2001; Broquet and Petit 2009). For example, genetic data have been used to measure dispersal rates and distances in seabirds (Peery et al. 2008), and to quantify dispersal bias between different age and sex classes of mammals (Fontanillas et al. 2004). Other studies have tested for sex-biased dispersal by contrasting sex-specific spatial patterns with spatial autocorrelation analysis, under the assumption that increased philopatry by one sex causes greater genetic similarity among neighboring same-sex individuals (Peakall et al. 2003; Coulon et al. 2006; Banks and Peakall 2012). Genetic data have also been used to assess dispersal pattern and kinship in mammals, for example: white-toothed shrew, *Crocidura russula* (Favre et al. 1997); brown long-eared bat, *Plecotus auritus* (Burland et al. 2001); lion, *Panthera leo* (Spong and Creel 2001); water vole, *Arvicola amphibious* (Telfer et al. 2003); banner-tailed kangaroo rats, *Dipodomys spectabilis* (Waser et al. 2006); and brown bear, *Ursus arctos* (Karamanlidis et al. 2021).

Ursids are generally solitarily, exhibit overlapping home ranges (Horner and Powell 1990; Mace and Waller 1997), and have a promiscuous mating system (Craighead et al. 1995; Schenk and Kovacs 1995; Steyaert et al. 2012). Dispersal in Ursids is usually male-biased, and males tend to disperse long distances, while female dispersal tends to be less common and they disperse comparatively short distances (Rogers 1987a; Støen et al. 2006; Zedrosser et al. 2007). Female philopatry may result in the formation of matrilineal assemblages, that is, a cluster of related females living in close proximity (Schenk et al. 1998; Støen et al. 2005; Moyer et al. 2006). Offspring in Ursids usually stay with their mother for 1–4 years, until they disperse: for example, American black bear, *U. americanus* (Rogers 1987a); brown bear, *U. arctos* (McLellan and Hovey 2001); and polar bear, *U. maritimus* (Ramsay and Stirling 1988).

Here, we investigated the patterns of natal dispersal in a solitary large mammal, the Asian black bear (*U. thibetanus*). It has been suggested that some Asian black bear females are philopatric (Kozakai et al. 2017), and that males disperse farther than females (Ohnishi and Osawa 2014); however, information on dispersal in this species is sparse and based on small sample sizes. Knowledge on dispersal in Asian black bears is also important for its management and conservation, because the species is commonly involved in human–bear conflicts, especially in Japan (Oi and Yamazaki 2006). We used genetic data and parentage analysis of management-killed bears and bears captured for research purposes to investigate sex-biased differences in the distance of natal dispersal. Based on patterns found in other mammals (Greenwood 1980; Pusey 1987), including other bear species (Rogers 1987a; McLellan and Hovey 2001; Zedrosser et al. 2007), we hypothesized that (i) males disperse farther from their natal home range compared to females. We further investigated the age of dispersal with spatial autocorrelation analysis, that is, the change in the correlation between pairwise genetic distance and geographic distance in each sex and age group (Costello et al. 2008; Roy et al. 2012; Baguette et al. 2013). We hypothesized that (ii) males disperse as subadults, and that females remain philopatric. We predicted that the spatial autocorrelation among males changes with increasing age due to dispersal, while the spatial autocorrelation among females does not change with increasing age due to philopatry. In addition, we investigated potential effects of hard mast productivity on dispersal because Asian black bears may temporarily leave their home range and travel long distances in the autumn of years with poor hard mast productivity (Ohnishi et al. 2011; Koike et al. 2012), which may accelerate the dispersal process.

## Materials and Methods

### Study area and data collection.

**Fig. 1.**
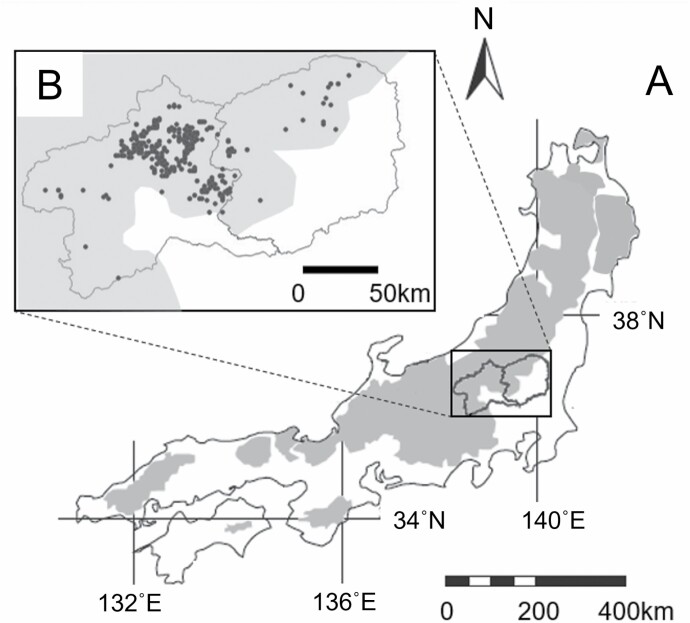
A) Distribution of Asian black bears (shaded) on central Honshu Island, Japan and in the prefectures studied from 2003 to 2018. B) Location of Gunma (to the west) and Tochigi (to the east) prefectures; black dots indicate the spatial distribution of capture and lethal removal locations of Asian black bears.

The study area was located in Gunma and Tochigi prefectures on central Honshu Island, Japan (Fig. 1). We used data from 550 bears (378 males, 172 females), of which 457 individuals were lethally removed due to nuisance control in Gunma prefecture (*n* = 435) in 2005–2018, and in Tochigi prefecture (*n* = 22) in 2003, 2004, and 2015. In addition, 93 individuals (56 males, 37 females) were captured with live traps as part of a research project in the Ashio–Nikko Mountains Range, which extended between the southern part of Tochigi Prefecture and the northern part of Gunma Prefecture, from 2003 to 2018 (e.g., Kozakai et al. 2011, 2017; Koike et al. 2012). The bears in these adjacent prefectures were part of the same population and the habitats were similar (Yoneda 2000). For all individuals in our data set, we obtained blood or tissue samples for genetic analysis as well as information on sex, age (based on tooth cementum layers; Grue and Jensen 1979), location, and date of capture or lethal removal. Trapping and handling of bears followed guidelines of the American Society of Mammalogists (Sikes et al. 2016). For more information on capture protocols, refer to the Guidelines for the Procedure of Obtaining Mammal Specimens as Approved by the Mammal Society of Japan (2009).

### Parentage analysis.

DNA was extracted from blood and tissue samples using a MagExtractor kit (Toyobo, Osaka, Japan). Genotypes at 14 DNA microsatellite loci (G1A, G1D, G10B, G10J, G10L, G10P, G10X, MSUT-1, MSUT-2, MSUT-6, MSUT-7, UarMU05, UarMU23, and UarMU50) were determined using polymerase chain reaction for all individuals (Paetkau et al. 1995, 1998; Kitahara et al. 2000). To ensure accuracy, genotypes were confirmed using the following method: for individuals that were captured only once, DNA extraction was performed twice. For individuals that had been captured more than once, blood was collected each time and DNA was extracted. PCR and genotyping were then performed from each DNA extraction solution, and only the loci that were confirmed to be the same genotype were used for subsequent analyses. Mother–offspring relationships were determined using CERVUS 3.07, which calculates log-likelihood ratio scores to estimate the most likely mother of the offspring (Kalinowski et al. 2007). Allele frequencies, heterozygosity, polymorphic information content (PIC), combined nonexclusion probabilities (for the first parent and second parent, parent pair, identity, and sibling identity), and the estimated null allele frequency (including mothers and offspring), which served as evaluation criteria for the accuracy of maternity determination, were calculated using the 550 unique genotypes of the captured bears. A positive log-likelihood ratio (LOD) score implies that a putative female is more likely to be the mother of a sampled individual compared to a randomly selected female. Statistical confidence (delta) was estimated for critical values at both strict (95%) and relaxed (80%) confidence levels on the basis of computer-simulated maternity inference using the allele frequencies of the 550 individuals.

Female Asian black bears in Japan may be sexually mature at the age of 3 and are commonly sexually mature at the age of 4 years (Katayama et al. 1996). We therefore defined individuals with an age difference of ≥3 years as potential mothers and offspring for the analysis of mother–offspring relationships in the software CERVUS 3.07 (Kalinowski et al. 2007).

### Natal dispersal distance.

Only adult mother–offspring pairs derived by parentage analysis were used for the estimation of dispersal distances. To evaluate the potential effects of mast failures on dispersal distances, we have carried out analyses (i) including all individuals and (ii) excluding individuals that were sampled during autumn of poor mast years which may have been captured or removed far from their home range. Therefore, for the second analysis we excluded mother–offspring pairs in which the mother or offspring was captured or removed during autumn (from September to November) of the poor hard mast years 2006, 2008, 2010, 2012, 2014, 2016, and 2018 (Kozakai et al. 2011; Gunma Prefecture 2018; Masaki et al. 2020).

We defined natal dispersal distance as the straight-line distance between the locations of an offspring and its mother. Adult female bears have stable interannual home ranges: Asian black bears (Yamamoto et al. 2012; Kozakai et al. 2017); American black bears (Costello 2010); and brown bears (Støen et al. 2005). Therefore, we used the capture/removal location of a mother as the natal home range center of an offspring. In the case of multiple captures of a mother, the first capture site when she was at least 3 years old was defined as the natal home range center of an offspring. A capture location was defined as the home range center of an offspring, because both male and female black bears generally have high home range fidelity once their home ranges are established (Costello 2010). In case of multiple captures of an offspring, the last capture site at the age when it was at least 3 years old was defined as the home range center of an offspring. We compared the estimated dispersal distances for each sex using two-sample *t*-tests. Also, we compared the estimated dispersal distances for (i) all individuals and (ii) excluding the individuals that were sampled during autumn of poor mast years using two-sample *t*-tests.

### Timing of natal dispersal with spatial autocorrelation analysis.

We conducted spatial autocorrelation analysis to estimate dispersal age using individual genotypes in GENALEX 6.5 (Peakall et al. 2003; Peakall and Smouse 2012) separately for males and females following the methods of Smouse and Peakall (1999) and Peakall et al. (2003). We calculated pairwise genetic and geographic distance matrices to determine spatial autocorrelation coefficients (*rc*) for males (*n* = 360) and females (*n* = 166) with available location data in Gunma Prefecture (Hardy and Vekemans 1999; Smouse and Peakall 1999). We focused in this analysis on Gunma Prefecture, because the sample size was higher and the bear sampling locations were geographically more concentrated compared to Tochigi Prefecture (Fig. 1). Pairwise genetic distances for microsatellite loci were calculated following Smouse and Peakall (1999). The spatial autocorrelation coefficient provides a measure of genetic similarity between pairs of individuals within a spatial distance class using random permutations. It ranges from −1 to +1, with a mean of zero, which indicates no autocorrelation (Hardy and Vekemans 1999; Smouse and Peakall 1999). Random permutations provide a 95% confidence interval (CI) around the null hypothesis of no spatial autocorrelation and a significant positive or negative genetic structure is reached when *rc* falls outside this CI (Smouse and Peakall 1999). For the same reason as in the natal dispersal distance analysis, for the second analysis we excluded individuals (66 males, 33 females) captured or removed during autumn of the poor mast years.

Based on geographic pairwise distances between individuals, we created 10 distance classes of 2 km each. We chose 2 km for the size of the distance bins because the average home range size (95% minimum convex polygon) of adult females from May to July is <10 km^2^ in part of our study area (Kozakai et al. 2011), which corresponds to a radius of <1.8 km. In addition, even the average annual home range size of adult females including autumn, when the home range of Asian black bears is larger than in spring and summer, is <20 km^2^ in several different regions and has a radius of <2.5 km (Koike and Hazumi 2008; Izumiyama and Mochizuki 2009; Yamamoto et al. 2012). We focused on the first distance class because if individuals start dispersing from their natal range, any differences in spatial autocorrelation between the age groups are expected to be most apparent in this class. To estimate the age of dispersal, we binned all individuals within a geographic distance class into age groups (<1, 1–1.5, 1.5–2, 2–3, 3–4, 4–5, 5–6, 6 years and older), and calculated pairwise *rc* between all individuals. Fractional age was calculated based on the reported date of death and 1 January as assumed date of birth. This date of birth was chosen for simplicity reasons and based on the observation that most captive Asian black bears give birth in January (Iibuchi et al. 2009). We included the age group 1.5 years to estimate the mean age of dispersal because it is assumed that Asian black bear offspring separate from their mother at 1–2 years old, based on other bear species (Rogers 1987a; McLellan and Hovey 2001; Van de Walle et al. 2018). We first calculated pairwise *rc* between all individuals, which is referred to as the age group ≥0. We then subsequently removed other age groups (<1, 1–1.5, 1.5–2, 2–3, 3–4, 4–5, 5–6, 6 years and older), starting with the youngest age group (<1), and recalculated pairwise *rc* between all remaining individuals until no significant positive *rc* values were found. The sample size of each age group (≥0, ≥1, ≥1.5, ≥2, ≥3, ≥4, ≥5, and ≥6) is shown in Appendix I. The youngest age group without a significant positive *rc* value at a given distance class within 2 km was considered as the age of dispersal. Results are presented as correlograms (plots of *rc* as a function of distance), with 95% CI about *rc* estimated by 1,000 bootstraps. Positive spatial autocorrelation was determined when the probability *P* to achieve a value greater than or equal to the observed *rc* was less than 0.05, as determined through 999 random permutations of the individual genotypes among the geographic locations.

## Results

### Natal dispersal distance.

**Fig. 2.**
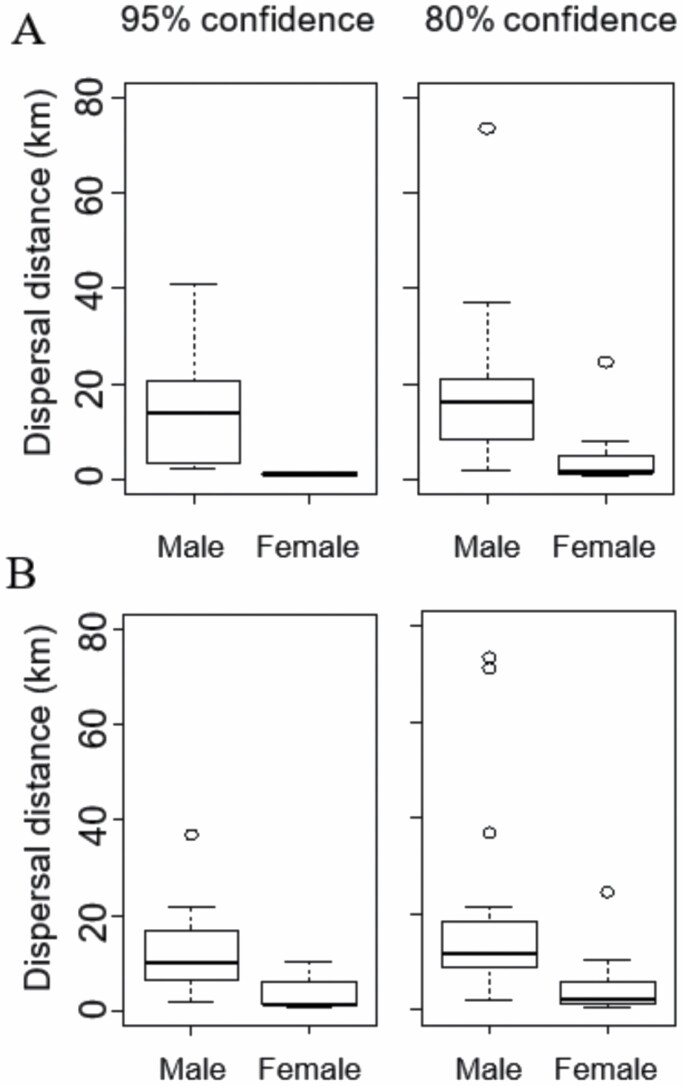
A) Dispersal distance of Asian black bears. The individuals whose mothers were identified at the 95% confidence level (*n* = 16; 11 males, 5 females) and 80% confidence level (*n* = 40; 26 males, 14 females). B) Dispersal distance of Asian black bears excluding individuals captured or removed during autumn in the poor hard mast years. The individuals whose mothers were identified at the 95% confidence level (*n* = 9; 6 males, 3 females) and 80% confidence level (*n* = 20; 10 males, 10 females). Boxes indicate the median and 25% and 75% quartiles, and whiskers show the smallest or largest values that are not outliers (open circles).

We identified the mothers of 25 individuals (15 males, 10 females) at a 95% confidence level, and the mothers of 56 individuals (33 males, 23 females) at an 80% confidence level (mean number of alleles = 8.571; mean observed heterozygosity = 0.655; mean expected heterozygosity = 0.708; mean PIC = 0.663; see also Appendices II and III). Removing individuals below the age of 3 resulted in a sample size of 16 individuals (11 males, 5 females) at the 95% confidence level, and 40 individuals (26 males, 14 females) at the 80% confidence level. To increase our sample size and because the results did not differ between 95% and 80% confidence levels (Fig. 2A), we continued with a confidence level of 80% for further analyses.

Males dispersed significantly farther than females (two-sample *t*-test, *P* < 0.05; Fig. 2A). The mean (± *SE*) and median (range) dispersal distance of males was 17.4 ± 3.5 km and 11.7 km (1.7–73.7 km), respectively. The mean and median dispersal distance of females was 4.8 ± 1.7 km and 2.2 km (0.4–24.4 km), respectively (Fig. 2A, Appendix IV). Based on a mean adult female home range radius of 1.8 km in our study area (Kozakai et al. 2011), 96% of the males and 50% of the females dispersed.

We repeated the same analysis as above after the removal of individuals captured during autumn in years with poor food availability. Analysis after the removal resulted in a sample size of 9 individuals (6 males, 3 females) at the 95% confidence level, and 20 individuals (10 males, 10 females) at the 80% confidence level. The results did not differ between 95% and 80% confidence levels, and males dispersed significantly farther than females (two-sample *t*-test, *P* < 0.05; Fig. 2B). The mean and median dispersal distance of males was 20.5 ± 6.7 km and 16.1 km (1.7–73.7 km), respectively. The mean and median dispersal distance of females was 4.7 ± 2.3 km and 1.5 km (0.4–24.4 km), respectively (Fig. 2B, Appendix IV). Based on a mean adult female home range radius of 1.8 km in our study area (Kozakai et al. 2011), 90% of the males and 40% of the females dispersed. We found no effect of mast failure on dispersal distances, that is, there was no significant difference in mean male and female dispersal distances when comparing distances during all years of our study period, which include years with good food conditions as well as years with mast failure, to distances in years with only good food conditions (two-sample *t*-test, male, *P* = 0.66; female, *P* = 0.98).

### Timing of natal dispersal.

**Fig. 3.**
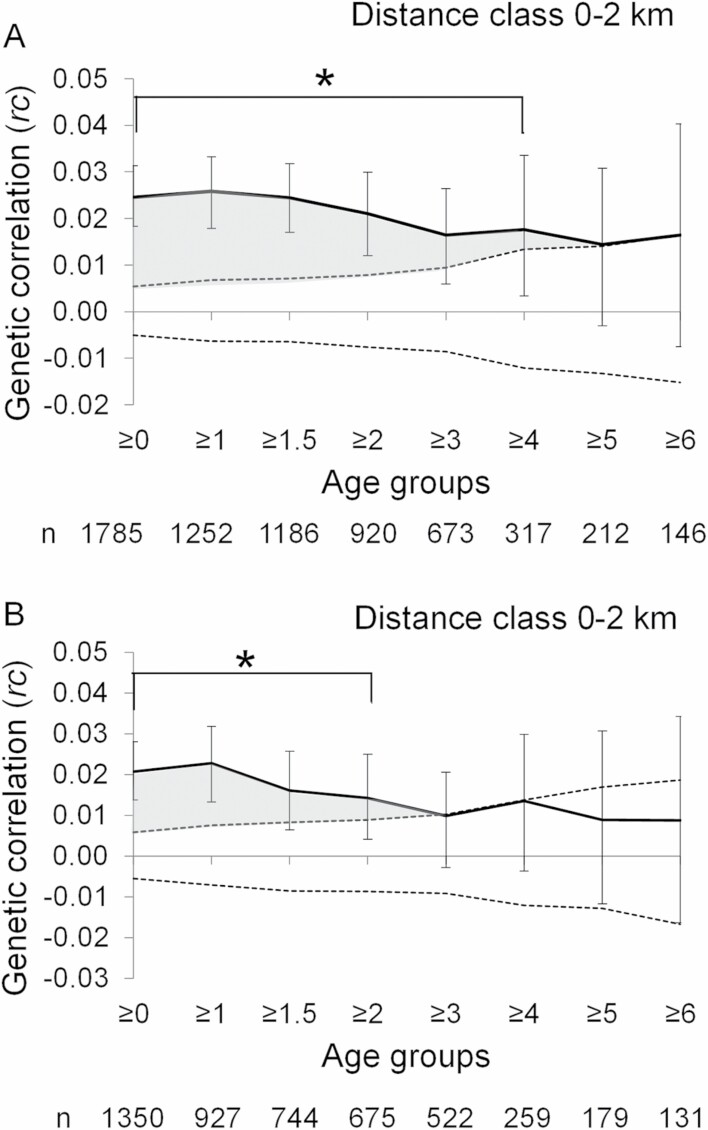
Relationship between geographic and genetic distance in male Asian black bears of A) all samples, and B) excluding individuals captured or removed during the autumn of poor hard mast years. The age group ≥0 represents the overall sample for each analysis. Solid lines with error bars represent the spatial autocorrelation coefficient *rc* and its 95% confidence interval. Horizontal dashed lines represent the 95% confidence interval of a random distribution of genotypes. The gray areas and asterisks (*) indicate significant genetic relationships (*P* < 0.05). The “*n*” sample size showed the number of pairwise comparisons, which analyzed the correlation between pairwise genetic distance and geographic distance.

**Fig. 4.**
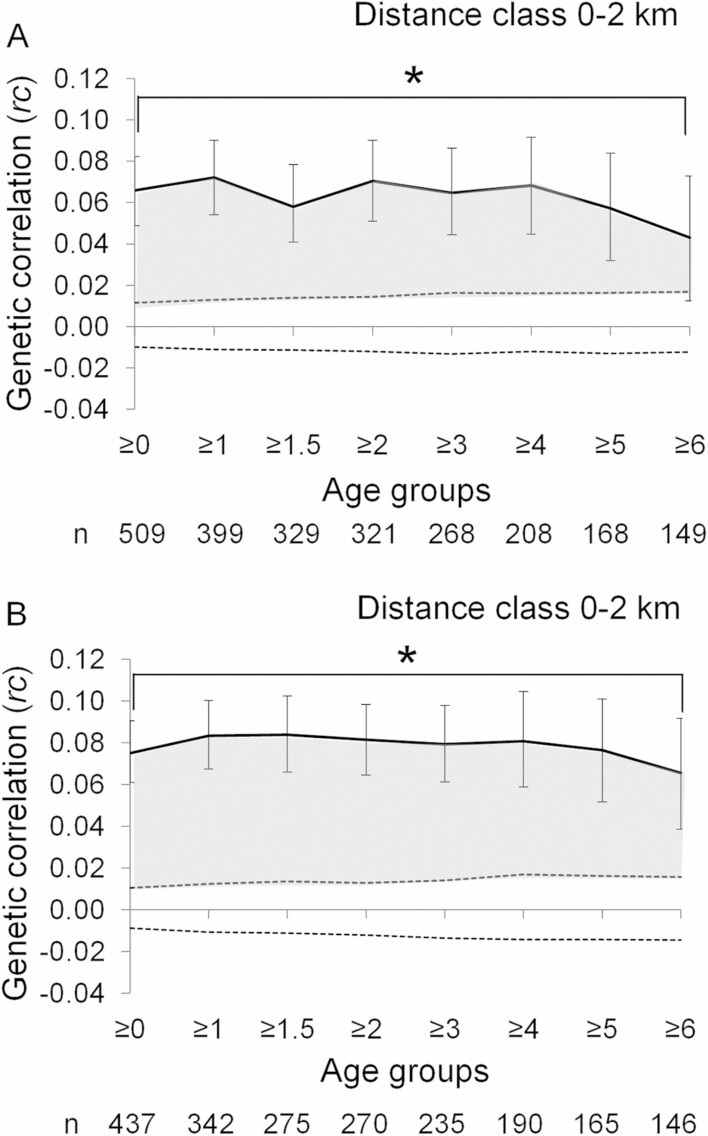
Relationship between geographic and genetic distance in female Asian black bears for A) all samples, and B) excluding individuals captured or removed during the autumn of poor hard mast years. The age group ≥0 represents the overall sample for each analysis. Solid lines with error bars represent the spatial autocorrelation coefficient *rc* and its 95% confidence interval. Horizontal dashed lines represent the 95% confidence interval of a random distribution of genotypes. The gray areas and asterisks (*) indicate significant genetic relationships (*P* < 0.05). The “*n*” sample size showed the number of pairwise comparisons, which analyzed the correlation between pairwise genetic distance and geographic distance.

In general, the relationship between genetic and geographic distances were more pronounced in males compared to females (Figs. 3 and 4). Banks and Peakall (2012) suggested that an appropriate bin size of spatial autocorrelation may be at or below the dispersal distance of the more philopatric sex. We choose 2 km as the appropriate bin size, because our results show that the median dispersal distance of females was 2.2 km.

In males, a significant genetic relationship among individuals within a distance of 2 km was observed for the age groups ≥0 to ≥4 years (Fig. 3, Supplementary Data SD1). After removal of individuals captured during the autumn of poor mast years, a significant genetic relationship within a distance of 2 km was observed for the age groups ≥0 to ≥2 years (Fig. 3). In females, a significant genetic relationship among individuals within 2 km was observed among all individuals in the age groups ≥0 to ≥6 years, both before and after removal of individuals captured during autumn of poor mast years (Fig. 4, Supplementary Data SD2).

## Discussion

### Natal dispersal distance.

Our results are generally consistent with male-biased dispersal and female-biased philopatry commonly reported in mammals (Greenwood 1980; Pusey 1987; Lawson Handley and Perrin 2007). Male-biased dispersal has also been reported in American black and brown bears (e.g., Rogers 1987a; McLellan and Hovey 2001; Støen et al. 2005). A similar pattern has been suggested for Asian black bears based on genetic analyses (Ishibashi and Saitoh 2004; Ohnishi and Osawa 2014); however, the results of these studies remained inconclusive. Our results suggest male-biased dispersal in Asian black bears. Based on median observed dispersal distances in our study (2.2 km for females and 11.7 km for males) and compared to the mean home range radius of GPS-collared adult female black bears in our study area (1.8 km; Kozakai et al. 2011), mature females likely establish their home range close to or overlapping with their natal range, whereas males disperse farther from their natal range.

The only other reported natal dispersal distances of Asian black bears were of three males that had dispersed 17 km, 25 km, and 41 km, respectively (Maita 1995; Ishida 2001; Yamazaki 2011). These distances, although observed in a different area, fall within the range observed in our study population (Maita 1995; Ishida 2001; Yamazaki 2011). The home range size of male Asian black bears has been estimated to vary from <5 km^2^ to >100 km^2^, and the average home range size is 30–60 km^2^ in Japan (Katayama 1999; Koike and Hazumi 2008; Izumiyama and Mochizuki 2009), which corresponds to a diameter of 6–9 km. The median dispersal distance of males in our study was 16.1 km, and almost all males (96%) dispersed the equivalent of at least one diameter of an adult male home range. Our results on dispersal distances are also supported by observations that the genetic correlation coefficients were significantly higher than expected within the 0–20 km distance class for males in other regions in Japan (Ohnishi and Osawa 2014).

Costello (2010) showed that the ‘floating’ period, that is, the period of natal dispersal before an individual settles into a permanent home range, in American black bears varied from a few months to a few years. In European brown bears, males settled into a home range approximately at the age of 4 years (Støen et al. 2006). Based on our result, 9 of 26 males used to estimate dispersal distances were 3 years old, and these males may not have established a permanent home range yet, which in turn may have an effect on the overall length of our dispersal estimates. In addition, the use of a capture/removal location as home range center of previously unknown individuals may affect dispersal distance estimates, which may be more pronounced in poor hard mast years when some bears move long distances in search of food (Ohnishi et al. 2011; Koike et al. 2012). Our results showed no effect of mast failure on dispersal distances, which also could be due to the ‘floating’ period (Costello 2010). For males whose home range has not yet been established, long-distance movement in years of poor hard mast may be part of the dispersal process, which could have resulted in no significant difference in mean dispersal distance.

Female bears commonly stay in or close to their mother’s home range and the formation of matrilineal assemblages has been shown in American black bears and brown bears (e.g., Støen et al. 2005; Moyer et al. 2006; Immell et al. 2014). Our results showed that about half of the females (50%) dispersed from their natal home range. In Scandinavian brown bears, 32%–46% of females dispersed from their natal home range (Støen et al. 2006), but almost no female dispersal has been reported in North America (e.g., McLellan and Hovey 2001). Støen et al. (2006) suggested that the high proportion of dispersing females in Scandinavia may be explained by the fact that the population was expanding and unoccupied areas were available at the edges of the population. The bear habitat in our study area is generally assumed to be saturated, as the distribution of populations covers the entire mountainous area assumed to be suitable for bears in this area (Japan Bear Network 2014). However, there are indications for regional variations in population density in this area (Gunma Prefecture 2017), which in turn suggests that female dispersal may be affected by variations of population density in relation to local or regional carrying capacities.

### Timing of dispersal.

The results from the spatial autocorrelation analysis of all samples suggest that males disperse at 5 years of age. However, after the exclusion of individuals captured/removed during poor mast years, our results show that the majority of males disperse already at 3 years of age. Mast failures may cause temporary long-distance movements to find more abundant food sources in Asian black bears (Ohnishi et al. 2011; Koike et al. 2012), and such movements may accelerate the dispersal process in young individuals. In contrast, our results suggest that mast failures do not advance the age of dispersal, likely because the factors driving the dispersal process are related to population structure rather than forage availability. Our results also suggest that some subadult males remain close to their natal range for some years after leaving it, and may even return to their natal range in poor mast years, presumably to look for food in a familiar area. The dispersal distance results, which showed that 1 of 26 males remained philopatric, could be due to the behavioral pattern of males. In several American black and brown bear populations, offspring separate from their mother at 1–2 years of age, and especially female offspring may remain within or close to the home range of the mother for the ensuing 1–2 years before ultimately dispersing (Rogers 1987a; McLellan and Hovey 2001; Van de Walle et al. 2018). Similarly, Asian black bear offspring may separate from their mother at 1–2 years old, but mostly disperse at 3 years of age.

Dispersal behavior can be influenced by the competitive ability of an individual (e.g., body size, condition, experience), especially in high-density populations (Mueller 1988). Ekman et al. (1999) suggested that this results in a competitive advantage of individuals delaying dispersal until reaching a certain threshold size or age. In addition, males likely take several years to complete dispersal, which may extend beyond their age at sexual maturity (which is at 2 or 3 years of age in Asian black bears in Japan; Komatsu et al. 1994). This pattern may be explained by subadult males remaining relatively close to their natal range, until they have reached a size that enables them to succeed in male–male competition for resources (e.g., Costello et al. 2008). Although no robust population density estimates exist for our study area, trend estimates suggest that the population is gradually increasing but that the distribution of bears has not changed significantly during this decade (Gunma Prefecture 2017). The number of management removals vary each year due to variations in hard mast productivity, and hard mast failures can cause intrusion of bears into residential areas (Oka et al. 2004; Katahira 2010). A similar pattern has also been observed in other species, where individuals that delayed dispersal obtained a competitive advantage for improved survival and reproduction: for example, Siberian jays, *Perisoreus infaustus* (Ekman et al. 1999); red wolves, *Canis rufus* (Sparkman et al. 2011); and Eurasian beavers, *Castor fiber* (Mayer et al. 2017). Our results support that dispersal is a gradual process that may take up to several years, and that delaying dispersal could be beneficial in a longer-term perspective (Weimerskirch 1992; Piper et al. 2015; Mayer et al. 2017).

Kin-related social structures and matrilineal assemblages have been documented in several mammals: for example, white-nosed coati, *Nasua narica* (Gompper and Wayne 1996); grey-sided voles, *Clethrionomys rufocanus* (Ishibashi et al. 1997), American black bears (Schenk et al. 1998); brown bears (Støen et al. 2005); and Asian black bears (Kozakai et al. 2017). A kin-related social structure in females may influence reproductive success and survival and, hence, population dynamics (Lambin and Krebs 1993). The spatial autocorrelation among females in our study population did not change with increasing age, suggesting related females form matrilineal assemblages, where members have more home range overlap than unrelated females. The formation of matrilineal assemblages is supported by results from GPS-collared bears in our study area showing that 11 closely related adult females (mothers, daughters, and sisters) lived within mean distances of 0.9–1.4 km (Kozakai et al. 2017). Kozakai et al. (2017) suggested that the benefits of matrilineal site fidelity especially during spring, summer, and the denning season could be key factors explaining the mechanism for the formation of matrilineal assemblages. The annual variation in food availability of Asian black bears is relatively small during spring and summer compared to variations in autumn (Koike 2010). Site fidelity likely results in improved foraging efficiency in a known and stable home range (Kozakai et al. 2017). Pinter-Wollman et al. (2009) reported that familiarity with a region improved foraging efficiency and reduced foraging time for African elephants, *Loxodonta africana*, which in turn may result in improved reproduction, which has also been suggested for American and Asian black bears (Kolenosky 1990; Kozakai et al. 2017). In addition, philopatric females may also gain benefits for reproduction and survival from associating with kin. Tolerance among kin can facilitate breeding success and survival in group-living mammals (Silk 2007; Clutton-Brock and Lukas 2012), and evidence for this has also been suggested in some large, solitary-living mammals: for example, American black bears (Rogers 1987b); and orangutan, *Pongo pygmaeus* (van Noordwijk et al. 2012). Due to potential benefits in reproduction and survival, philopatry is likely more attractive for subadult female Asian black bears compared to dispersal (see also Gundersen and Andreassen 1998).

Banks and Peakall (2012) reported that changes in the size of the distance bins can affect the performance of the spatial autocorrelation approach for dispersal, because the outcomes of spatial autocorrelation analyses reflect the interplay between sample size and the unknown extent of the structure (Peakall et al. 2003; Smouse et al. 2008). They suggested that the optimal distance class should closely correspond with the mean dispersal distance of the less dispersive sex (Banks and Peakall 2012). We used distance bins of 2 km based on adult female home range size and dispersal distances and found evidence for sex-biased dispersal. However, our sample sizes decreased with increasing minimum age of the age groups, that is, the power and significance of the spatial autocorrelation is lower in older age groups. Although we detected sex-biased dispersal in older age groups, analyses with larger sample size could assess the effect of the sample size differences.

In conclusion, we found evidence for male-biased dispersal in our study population. Dispersal in Asian black bears is a gradual process, and they gradually disperse farther away from their natal range over time. Most males disperse at age 3; however, young and inexperienced males may temporarily return to their natal range during poor mast years, likely to search for food in a familiar area. We also examined the effect of mast failure on dispersal. We found that mast failure did not affect dispersal distances or age, rather only temporary movements, which suggests that dispersal is driven by population-structure processes rather than fluctuations in food resources. During poor mast years, bears are commonly killed as nuisance animals, especially young males. In general, the proportion of young bears among killed nuisance bears has been increasing in more recent years (Anezaki 2019). Based on our findings, it is likely that many bears in the process of dispersal are either killed or captured as nuisance animals in years with unfavorable environmental conditions. These management actions may have long-term effects on population structure, which requires further attention from wildlife management authorities as well as researchers.

## Supplementary Data

Supplementary data are available at Journal of Mammalogy online.

**Supplementary Data SD1.**—Relationship between geographic and genetic distance in male Asian black bears for all samples. The age group ≥0 represents the overall sample for each analysis. Solid lines with error bars represent the spatial autocorrelation coefficient *rc* and its 95% confidence interval. Horizontal dashed lines represent the 95% confidence interval of a random distribution of genotypes.

**Supplementary Data SD2.**—Relationship between geographic and genetic distance in female Asian black bears for all samples. The age group ≥0 represents the overall sample for each analysis. Solid lines with error bars represent the spatial autocorrelation coefficient *rc* and its 95% confidence interval. Horizontal dashed lines represent the 95% confidence interval of a random distribution of genotypes.

gyac118_suppl_Supplementary_Material_SD1

gyac118_suppl_Supplementary_Material_SD2

## Notes

Submitted 7 September 2020. Accepted 18 November 2022.

## Appendix I

The sample size of each age group for spatial autocorrelation analysis in Asian black bear. The age group ≥0 means the group with a minimum age of 0 years, ≥1 means the group with a minimum age of 1 year. “All” means the sample size of each age group with all samples, and “Mast” means the sample size of each age group that excluded individuals captured or removed during autumn in the poor mast years.

**Table:**

Age groups									
		≥0	≥1	≥1.5	≥2	≥3	≥4	≥5	≥6
Male	All	360	291	279	252	214	129	92	70
	Mast	294	235	219	202	174	101	69	53
Female	All	166	141	133	128	111	85	63	52
	Mast	130	107	99	95	82	63	47	38

## Appendix II

List of estimated mother–offspring pairs in Asian black bears based on bears captured for scientific purposes or removed for management reasons on central Honshu Island, Japan, 2003–2018. “Offspring ID” shows individuals whose mothers were identified genetically (“mother ID”). “Reproductive age” shows the age at which a mother gave birth to an offspring. In the column “Pair confidence,” asterisk (*) indicates a 95% confidence level (CI) and plus (+) indicates an 80% CI of the results of the genetic parentage analysis.

**Table:**

Offspring ID	Mother ID	Pair confidence	Offspring sex	Offspring age	Reproductive age	Capture/removal year of offspring	Capture/removal year of mother
AF06	FB70	+	F	9	6	2011	2004/2010
AF16	AF23	*	F	7	4	2013	2008/2011
AF18	AF12	+	F	9	5	2015	2007
AF33	AF53	*	F	1	15	2010	2012
AF35	AF23	+	F	8	7	2017	2008/2011
AF45	AF09	+	F	8	4	2016	2006/2008/2013
AF55	AF60	*	F	7	8	2017	2012
AF56	AF30	*	F	5	5	2012	2009
AF81	AF16	*	F	1	10	2017	2008/2009/2013
AM25	AF24	*	M	3	6	2011	2008/2009
AM68	AF49	*	M	7	4	2017	2011
AM77	AF09	*	M	2	10	2016	2006/2008/2013
GM052	GM071	*	F	6	5	2009	2010
GM079	GM048	+	F	3	NA	2010	2009
GM082	GM081	+	F	0	5	2010	2010
GM083	GM081	*	F	0	5	2010	2010
GM093	GM080	+	M	NA	NA	2011	2010
GM100	GM080	*	M	4	8	2010	2010
GM107	FB74	+	M	4	10	2010	2003/2007
GM117	GM077	*	M	15	NA	2010	2010
GM142	GM071	*	F	6	8	2012	2010
GM161	GM170	+	M	1	6	2012	2012
GM169	AF24	+	M	5	5	2012	2008/2009
GM171	GM170	+	F	3	4	2012	2012
GM175	GM30	+	M	8	NA	2012	2008
GM18	GM065	+	F	NA	NA	2008	2010
GM190	GM070	+	M	NA	NA	2013	2010
GM196	GM095	*	M	2	NA	2013	2011
GM20	GM152	*	M	NA	NA	2008	2012
GM209	AF45	+	F	1	5	2014	2010/2012/2016
GM212	GM174	+	M	3	3	2014	2012
GM235	GM186	+	M	1	5	2014	2013
GM243	GM242	+	M	3	6	2014	2014
GM249	GM170	+	M	3	6	2014	2012
GM257	GM075	+	M	4	6	2014	2010
GM258	GM090	*	M	4	8	2014	2011
GM305	GM179	*	M	NA	NA	2015	2013
GM330	FB74	+	M	4	16	2016	2003/2007
GM333	GM282	*	F	3	3	2016	2015
GM334	GM309	*	M	2	NA	2016	2015
GM337	GM219	*	M	3	NA	2016	2014
GM363	GM302	+	F	2	3	2016	2015
GM369	GM071	*	M	3	9	2010	2010
GM371	GM219	+	M	1	NA	2010	2014
GM372	GM219	+	F	1	NA	2010	2014
GM373	GM088	+	M	4	7	2010	2010
GM379	GM231	*	M	1	3	2013	2014
GM387	GM142	*	M	3	6	2015	2012
GM431	GM327	+	M	NA	NA	2017	2016
GM447	GM365	+	M	3	3	2018	2016
GM449	AF16	+	F	2	10	2018	2008/2009/2013
GM457	GM180	+	F	6	3	2018	2013
GM461	AF49	*	F	1	11	2018	2011
TC13	GM077	+	M	3	NA	2004	2010
TC19	GM219	+	M	4	NA	2004	2014
TC22	GM282	*	M	NA	NA	2015	2015

## Appendix III

Summary statistics results for 14 microsatellite loci in Asian black bears on central Honshu Island, Japan, which were captured/removed in 2003–2018 (*n* = 553). Observed heterozygosity (*H*_Obs_), expected heterozygosity (*H*_Exp_), average nonexclusion probability for the first parent (NE-1P), average nonexclusion probability for the second parent (NE-2P), average nonexclusion probability for identity of two unrelated individuals (NE-I), average nonexclusion probability for identity of two siblings (NE-SI), and estimated null allele frequency (*F*(Null)) are shown.

**Table:**

Locus	*H*_Obs_	*H*_Exp_	PIC	NE-1P	NE-2P	NE-I	NE-SI	*F*(Null)
G1A	0.659	0.728	0.686	0.677	0.501	0.116	0.415	0.0491
G1D	0.530	0.579	0.514	0.825	0.681	0.242	0.521	0.0441
G10B	0.727	0.782	0.754	0.586	0.408	0.075	0.378	0.0364
G10J	0.643	0.688	0.642	0.727	0.552	0.143	0.442	0.0376
G10L	0.566	0.631	0.566	0.795	0.643	0.201	0.485	0.0545
G10P	0.513	0.565	0.492	0.840	0.707	0.262	0.533	0.0478
G10X	0.670	0.697	0.655	0.704	0.528	0.133	0.435	0.0206
MSUT-1	0.695	0.721	0.688	0.671	0.488	0.110	0.417	0.0159
MSUT-2	0.688	0.785	0.753	0.594	0.415	0.077	0.377	0.0648
MSUT-6	0.546	0.631	0.559	0.801	0.654	0.208	0.487	0.0710
MSUT--7	0.763	0.806	0.780	0.554	0.377	0.063	0.363	0.0266
UarMU05	0.771	0.814	0.791	0.535	0.360	0.057	0.358	0.0262
UarMU23	0.794	0.821	0.804	0.504	0.331	0.048	0.352	0.0175
UarMU50	0.606	0.666	0.602	0.759	0.602	0.176	0.461	0.0500

## Appendix IV

The list of dispersal distances in male (*n* = 26) and female (*n* = 14) Asian black bears on central Honshu Island, Japan, which were captured/removed in 2003–2018. The offspring ID shows the individuals whose mothers were identified at >80% confidence level of the parentage analysis. The checkmark on Poor mast shows the individual or the mother was captured/removed during autumn in years with poor food availability. We removed individuals below the age of 3, as they may still be with their mothers.

**Table:**

Offspring ID (male)	Dispersal distance (m)	Age	Poor mast	Offspring ID (female)	Dispersal distance (m)	Age	Poor mast
AM25	1,659	3		AF06	3,771	9	
AM68	2,787	7		AF16	1,121	7	
GM093	5,650	NA	√	AF18	1,381	9	
GM190	8,971	NA	√	AF35	1,121	8	
GM212	4,979	3	√	AF45	1,532	8	
GM305	36,873	NA		AF55	987	7	
GM330	20,943	4		AF56	405	5	
GM337	18,254	3		GM052	10,213	6	√
GM369	8,974	3	√	GM079	923	3	√
GM387	9,976	3		GM142	5,981	6	√
GM431	17,264	NA		GM171	2,773	3	√
GM447	8,285	3		GM18	24,389	NA	
TC13	71,357	3	√	GM333	7,659	3	
TC19	73,699	4		GM457	4,756	6	
TC22	15,016	NA					
GM100	21,525	4	√				
GM20	10,056	NA	√				
GM107	17,596	4	√				
GM117	8,534	15	√				
GM169	20,678	5	√				
GM175	12,071	8	√				
GM243	11,318	3	√				
GM249	9,648	3	√				
GM257	15,908	4	√				
GM258	4,309	4	√				
GM373	15,369	4	√				
Mean distance ± *SE* (male)	17.4 ± 3.5 km			Mean distance ± *SE* (female)	4.8 ± 1.7 km		
