# Supplementary material for: Timing and distance of natal dispersal in Asian black bears
Source: J Mammal. 2023 Feb 14;104(2):265–78. doi: 10.1093/jmammal/gyac118 (PMC10075337; doi:10.1093/jmammal/gyac118)

Supplementary Data SD2.—Relationship between geographic and genetic distance in female Asian black bears for all sample, which were captured for scientific purposes or removed for management reasons on central Honshu Island, Japan, 2003–2018. The age group ≥0 represents the overall sample for each analysis. Solid lines with error bars represent the spatial autocorrelation coefficient *rc* and its 95% confidence interval. Horizontal dashed lines represent the 95% confidence interval of a random distribution of genotypes.

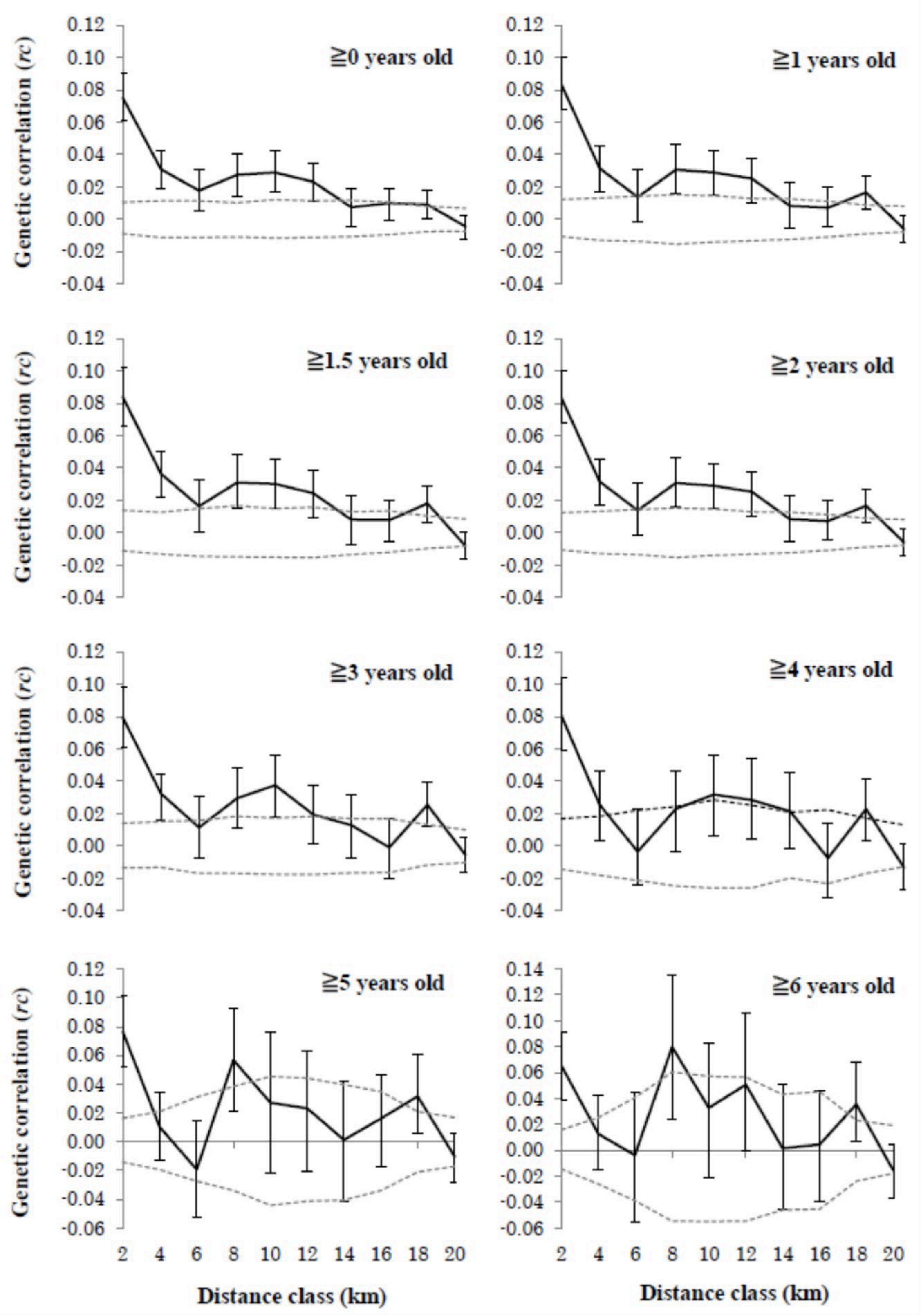

Supplement: gyac118_suppl_Supplementary_Material_SD2 [file gyac118_suppl_supplementary_material_sd2.pdf]
